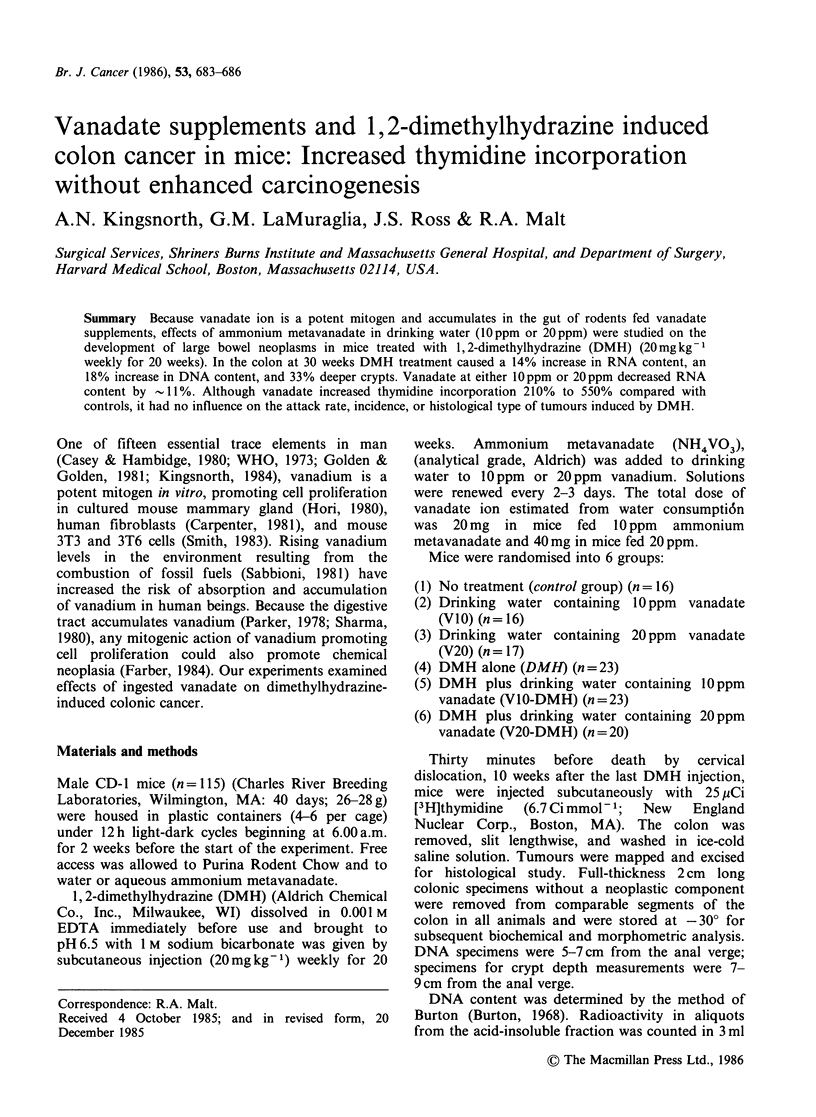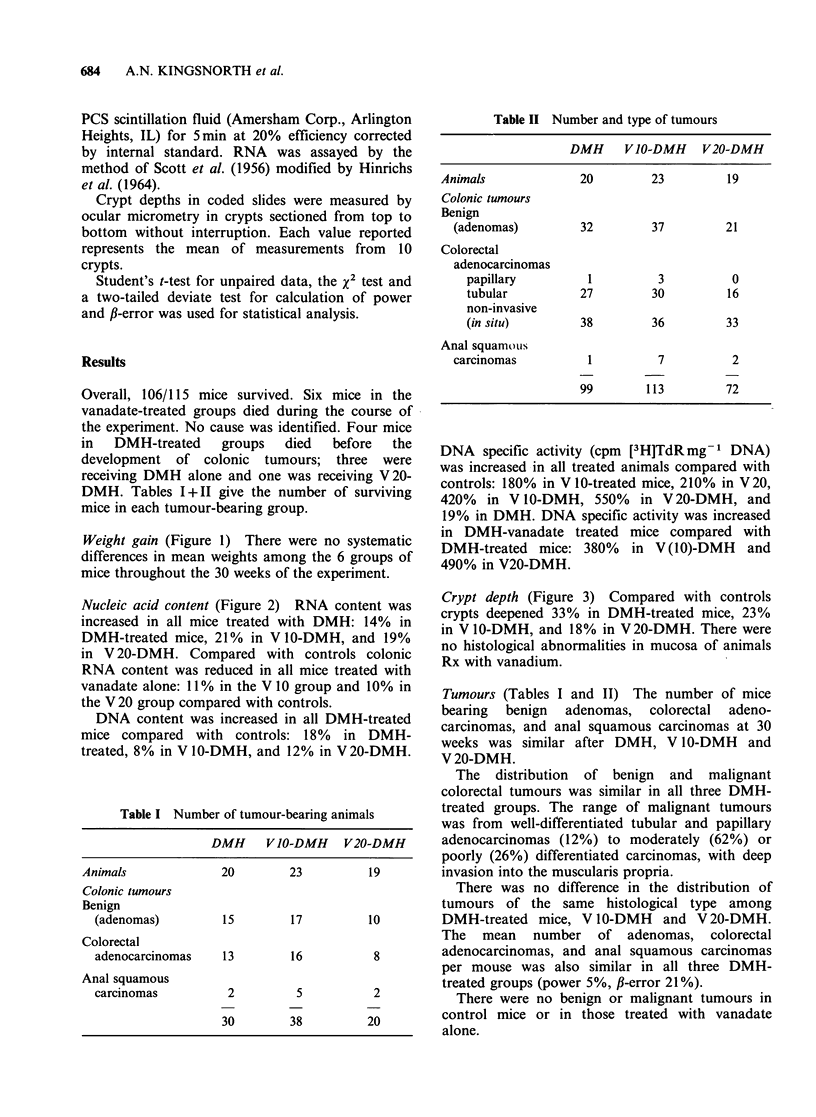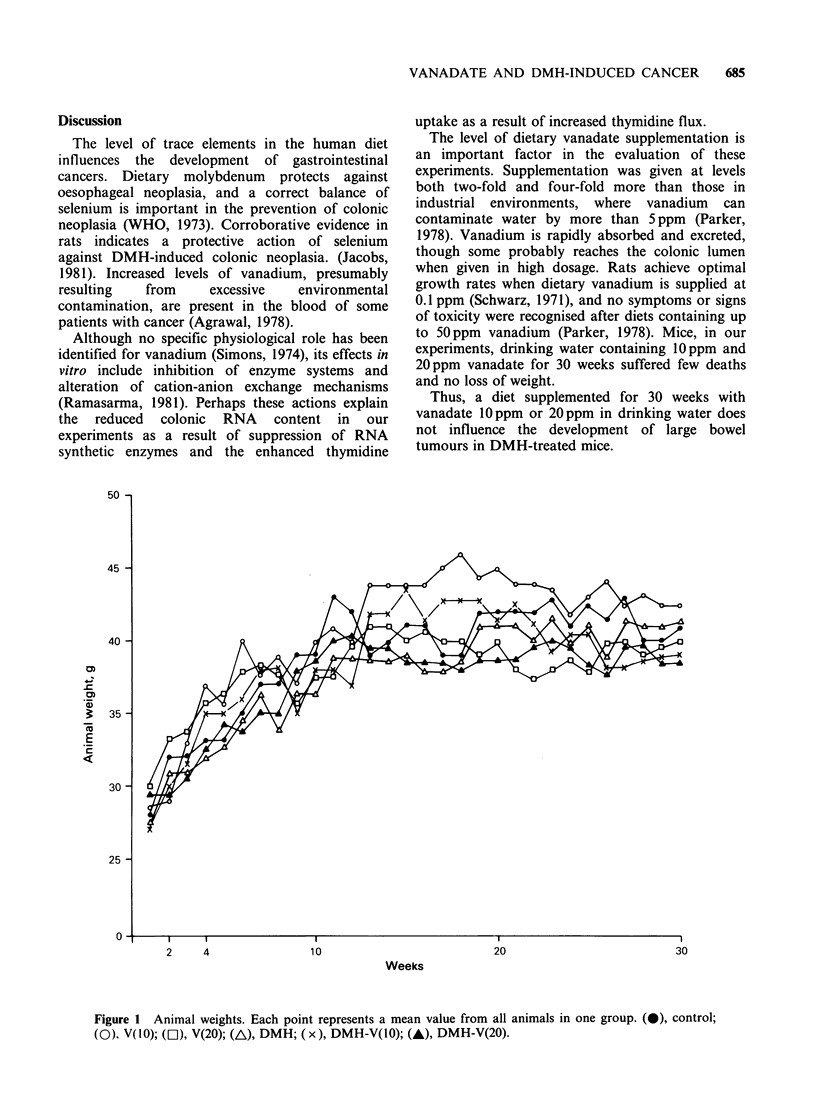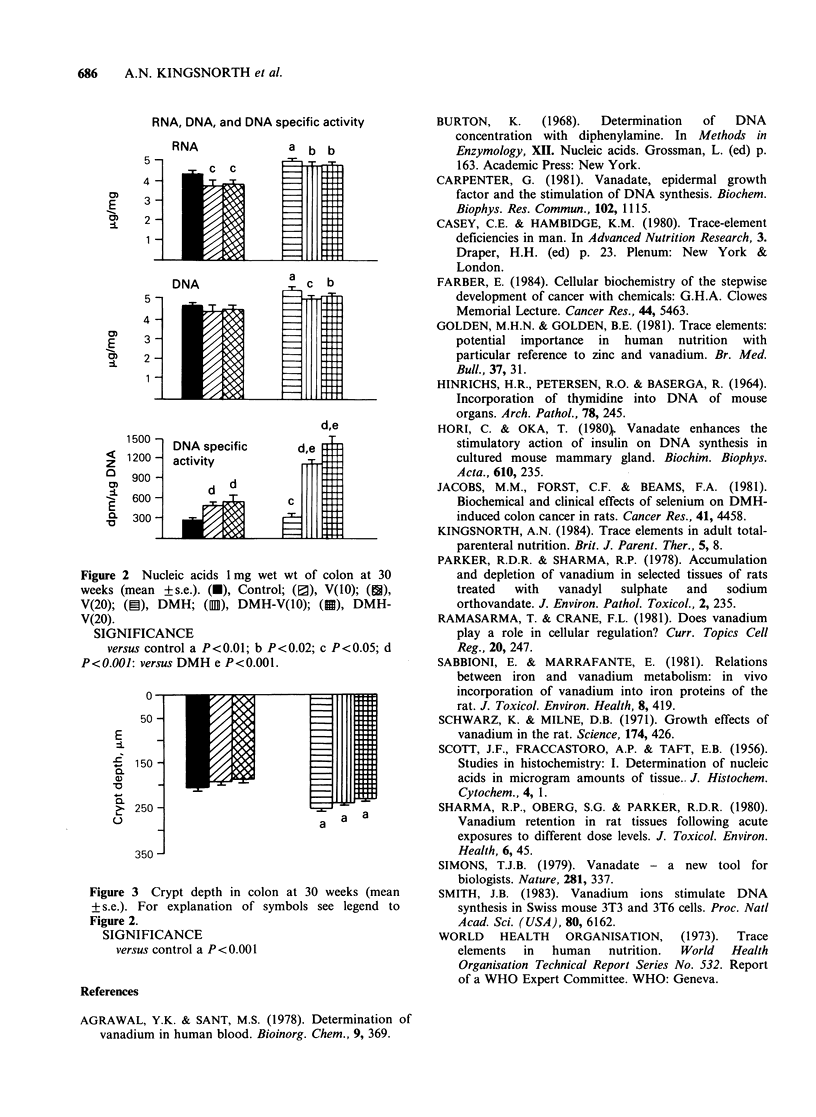# Vanadate supplements and 1,2-dimethylhydrazine induced colon cancer in mice: increased thymidine incorporation without enhanced carcinogenesis.

**DOI:** 10.1038/bjc.1986.112

**Published:** 1986-05

**Authors:** A. N. Kingsnorth, G. M. LaMuraglia, J. S. Ross, R. A. Malt

## Abstract

Because vanadate ion is a potent mitogen and accumulates in the gut of rodents fed vanadate supplements, effects of ammonium metavanadate in drinking water (10 ppm or 20 ppm) were studied on the development of large bowel neoplasms in mice treated with 1,2-dimethylhydrazine (DMH) (20 mg kg-1 weekly for 20 weeks). In the colon at 30 weeks DMH treatment caused a 14% increase in RNA content, an 18% increase in DNA content, and 33% deeper crypts. Vanadate at either 10 ppm or 20 ppm decreased RNA content by approximately 11%. Although vanadate increased thymidine incorporation 210% to 550% compared with controls, it had no influence on the attack rate, incidence, or histological type of tumours induced by DMH.


*Br. J. Cancer* (1986), **53**, 683–686

### Vanadate supplements and 1,2-dimethylhydrazine induced colon cancer in mice: Increased thymidine incorporation without enhanced carcinogenesis

A.N. Kingsnorth, G.M. LaMuraglia, J.S. Ross & R.A. Malt

*Surgical Services, Shriners Burns Institute and Massachusetts General Hospital, and Department of Surgery, Harvard Medical School, Boston, Massachusetts 02114, USA.*

**Summary** Because vanadate ion is a potent mitogen and accumulates in the gut of rodents fed vanadate supplements, effects of ammonium metavanadate in drinking water (10 ppm or 20 ppm) were studied on the development of large bowel neoplasms in mice treated with 1,2-dimethylhydrazine (DMH) (20 mg kg$^{-1}$ weekly for 20 weeks). In the colon at 30 weeks DMH treatment caused a 14% increase in RNA content, an 18% increase in DNA content, and 33% deeper crypts. Vanadate at either 10 ppm or 20 ppm decreased RNA content by ~11%. Although vanadate increased thymidine incorporation 210% to 550% compared with controls, it had no influence on the attack rate, incidence, or histological type of tumours induced by DMH.

One of fifteen essential trace elements in man (Casey & Hambidge, 1980; WHO, 1973; Golden & Golden, 1981; Kingsnorth, 1984), vanadium is a potent mitogen *in vitro*, promoting cell proliferation in cultured mouse mammary gland (Hori, 1980), human fibroblasts (Carpenter, 1981), and mouse 3T3 and 3T6 cells (Smith, 1983). Rising vanadium levels in the environment resulting from the combustion of fossil fuels (Sabbioni, 1981) have increased the risk of absorption and accumulation of vanadium in human beings. Because the digestive tract accumulates vanadium (Parker, 1978; Sharma, 1980), any mitogenic action of vanadium promoting cell proliferation could also promote chemical neoplasia (Farber, 1984). Our experiments examined effects of ingested vanadate on dimethylhydrazine-induced colonic cancer.

### Materials and methods

Male CD-1 mice ($n=115$) (Charles River Breeding Laboratories, Wilmington, MA: 40 days; 26–28 g) were housed in plastic containers (4–6 per cage) under 12 h light-dark cycles beginning at 6.00 a.m. for 2 weeks before the start of the experiment. Free access was allowed to Purina Rodent Chow and to water or aqueous ammonium metavanadate.

1,2-dimethylhydrazine (DMH) (Aldrich Chemical Co., Inc., Milwaukee, WI) dissolved in 0.001 M EDTA immediately before use and brought to pH 6.5 with 1 M sodium bicarbonate was given by subcutaneous injection (20 mg kg$^{-1}$) weekly for 20 weeks. Ammonium metavanadate ($NH_4VO_3$), (analytical grade, Aldrich) was added to drinking water to 10 ppm or 20 ppm vanadium. Solutions were renewed every 2–3 days. The total dose of vanadate ion estimated from water consumptiön was 20 mg in mice fed 10 ppm ammonium metavanadate and 40 mg in mice fed 20 ppm.

Mice were randomised into 6 groups:

(1) No treatment (*control* group) ($n=16$)

(2) Drinking water containing 10 ppm vanadate (V10) ($n=16$)

(3) Drinking water containing 20 ppm vanadate (V20) ($n=17$)

(4) DMH alone (*DMH*) ($n=23$)

(5) DMH plus drinking water containing 10 ppm vanadate (V10-DMH) ($n=23$)

(6) DMH plus drinking water containing 20 ppm vanadate (V20-DMH) ($n=20$)

Thirty minutes before death by cervical dislocation, 10 weeks after the last DMH injection, mice were injected subcutaneously with 25 $\mu$Ci [$^3$H]thymidine (6.7 Ci mmol$^{-1}$; New England Nuclear Corp., Boston, MA). The colon was removed, slit lengthwise, and washed in ice-cold saline solution. Tumours were mapped and excised for histological study. Full-thickness 2 cm long colonic specimens without a neoplastic component were removed from comparable segments of the colon in all animals and were stored at −30° for subsequent biochemical and morphometric analysis. DNA specimens were 5–7 cm from the anal verge; specimens for crypt depth measurements were 7–9 cm from the anal verge.

DNA content was determined by the method of Burton (Burton, 1968). Radioactivity in aliquots from the acid-insoluble fraction was counted in 3 ml

Correspondence: R.A. Malt.
Received 4 October 1985; and in revised form, 20 December 1985

© The Macmillan Press Ltd., 1986

PCS scintillation fluid (Amersham Corp., Arlington Heights, IL) for 5 min at 20% efficiency corrected by internal standard. RNA was assayed by the method of Scott *et al.* (1956) modified by Hinrichs *et al.* (1964).

Crypt depths in coded slides were measured by ocular micrometry in crypts sectioned from top to bottom without interruption. Each value reported represents the mean of measurements from 10 crypts.

Student's *t*-test for unpaired data, the $\chi^2$ test and a two-tailed deviate test for calculation of power and $\beta$-error was used for statistical analysis.

### Results

Overall, 106/115 mice survived. Six mice in the vanadate-treated groups died during the course of the experiment. No cause was identified. Four mice in DMH-treated groups died before the development of colonic tumours; three were receiving DMH alone and one was receiving V20-DMH. Tables I+II give the number of surviving mice in each tumour-bearing group.

*Weight gain* (Figure 1) There were no systematic differences in mean weights among the 6 groups of mice throughout the 30 weeks of the experiment.

*Nucleic acid content* (Figure 2) RNA content was increased in all mice treated with DMH: 14% in DMH-treated mice, 21% in V10-DMH, and 19% in V20-DMH. Compared with controls colonic RNA content was reduced in all mice treated with vanadate alone: 11% in the V10 group and 10% in the V20 group compared with controls.

DNA content was increased in all DMH-treated mice compared with controls: 18% in DMH-treated, 8% in V10-DMH, and 12% in V20-DMH.

**Table I** Number of tumour-bearing animals

| | *DMH* | *V10-DMH* | *V20-DMH* |
|---|---|---|---|
| *Animals* | 20 | 23 | 19 |
| *Colonic tumours* | | | |
| Benign (adenomas) | 15 | 17 | 10 |
| Colorectal adenocarcinomas | 13 | 16 | 8 |
| Anal squamous carcinomas | 2 | 5 | 2 |
| | 30 | 38 | 20 |

**Table II** Number and type of tumours

| | *DMH* | *V10-DMH* | *V20-DMH* |
|---|---|---|---|
| *Animals* | 20 | 23 | 19 |
| *Colonic tumours* | | | |
| Benign (adenomas) | 32 | 37 | 21 |
| Colorectal adenocarcinomas | | | |
| papillary | 1 | 3 | 0 |
| tubular | 27 | 30 | 16 |
| non-invasive (*in situ*) | 38 | 36 | 33 |
| Anal squamous carcinomas | 1 | 7 | 2 |
| | 99 | 113 | 72 |

DNA specific activity (cpm [$^3$H]TdR mg$^{-1}$ DNA) was increased in all treated animals compared with controls: 180% in V10-treated mice, 210% in V20, 420% in V10-DMH, 550% in V20-DMH, and 19% in DMH. DNA specific activity was increased in DMH-vanadate treated mice compared with DMH-treated mice: 380% in V(10)-DMH and 490% in V20-DMH.

*Crypt depth* (Figure 3) Compared with controls crypts deepened 33% in DMH-treated mice, 23% in V10-DMH, and 18% in V20-DMH. There were no histological abnormalities in mucosa of animals Rx with vanadium.

*Tumours* (Tables I and II) The number of mice bearing benign adenomas, colorectal adenocarcinomas, and anal squamous carcinomas at 30 weeks was similar after DMH, V10-DMH and V20-DMH.

The distribution of benign and malignant colorectal tumours was similar in all three DMH-treated groups. The range of malignant tumours was from well-differentiated tubular and papillary adenocarcinomas (12%) to moderately (62%) or poorly (26%) differentiated carcinomas, with deep invasion into the muscularis propria.

There was no difference in the distribution of tumours of the same histological type among DMH-treated mice, V10-DMH and V20-DMH. The mean number of adenomas, colorectal adenocarcinomas, and anal squamous carcinomas per mouse was also similar in all three DMH-treated groups (power 5%, $\beta$-error 21%).

There were no benign or malignant tumours in control mice or in those treated with vanadate alone.

**Discussion**

The level of trace elements in the human diet influences the development of gastrointestinal cancers. Dietary molybdenum protects against oesophageal neoplasia, and a correct balance of selenium is important in the prevention of colonic neoplasia (WHO, 1973). Corroborative evidence in rats indicates a protective action of selenium against DMH-induced colonic neoplasia. (Jacobs, 1981). Increased levels of vanadium, presumably resulting from excessive environmental contamination, are present in the blood of some patients with cancer (Agrawal, 1978).

Although no specific physiological role has been identified for vanadium (Simons, 1974), its effects *in vitro* include inhibition of enzyme systems and alteration of cation-anion exchange mechanisms (Ramasarma, 1981). Perhaps these actions explain the reduced colonic RNA content in our experiments as a result of suppression of RNA synthetic enzymes and the enhanced thymidine uptake as a result of increased thymidine flux.

The level of dietary vanadate supplementation is an important factor in the evaluation of these experiments. Supplementation was given at levels both two-fold and four-fold more than those in industrial environments, where vanadium can contaminate water by more than 5 ppm (Parker, 1978). Vanadium is rapidly absorbed and excreted, though some probably reaches the colonic lumen when given in high dosage. Rats achieve optimal growth rates when dietary vanadium is supplied at 0.1 ppm (Schwarz, 1971), and no symptoms or signs of toxicity were recognised after diets containing up to 50 ppm vanadium (Parker, 1978). Mice, in our experiments, drinking water containing 10 ppm and 20 ppm vanadate for 30 weeks suffered few deaths and no loss of weight.

Thus, a diet supplemented for 30 weeks with vanadate 10 ppm or 20 ppm in drinking water does not influence the development of large bowel tumours in DMH-treated mice.

**Figure 1** Animal weights. Each point represents a mean value from all animals in one group. (●), control; (○), V(10); (□), V(20); (△), DMH; (×), DMH-V(10); (▲), DMH-V(20).

**Figure 2** Nucleic acids 1 mg wet wt of colon at 30 weeks (mean ±s.e.). (■), Control; (▨), V(10); (▩), V(20); (▤), DMH; (▥), DMH-V(10); (▦), DMH-V(20).

SIGNIFICANCE

*versus* control a $P<0.01$; b $P<0.02$; c $P<0.05$; d $P<0.001$: *versus* DMH e $P<0.001$.

**Figure 3** Crypt depth in colon at 30 weeks (mean ±s.e.). For explanation of symbols see legend to **Figure 2**.

SIGNIFICANCE

*versus* control a $P<0.001$

**References**

AGRAWAL, Y.K. & SANT, M.S. (1978). Determination of vanadium in human blood. *Bioinorg. Chem.*, **9**, 369.

BURTON, K. (1968). Determination of DNA concentration with diphenylamine. In *Methods in Enzymology*, **XII.** Nucleic acids. Grossman, L. (ed) p. 163. Academic Press: New York.

CARPENTER, G. (1981). Vanadate, epidermal growth factor and the stimulation of DNA synthesis. *Biochem. Biophys. Res. Commun.*, **102**, 1115.

CASEY, C.E. & HAMBIDGE, K.M. (1980). Trace-element deficiencies in man. In *Advanced Nutrition Research*, **3.** Draper, H.H. (ed) p. 23. Plenum: New York & London.

FARBER, E. (1984). Cellular biochemistry of the stepwise development of cancer with chemicals: G.H.A. Clowes Memorial Lecture. *Cancer Res.*, **44**, 5463.

GOLDEN, M.H.N. & GOLDEN, B.E. (1981). Trace elements: potential importance in human nutrition with particular reference to zinc and vanadium. *Br. Med. Bull.*, **37**, 31.

HINRICHS, H.R., PETERSEN, R.O. & BASERGA, R. (1964). Incorporation of thymidine into DNA of mouse organs. *Arch. Pathol.*, **78**, 245.

HORI, C. & OKA, T. (1980). Vanadate enhances the stimulatory action of insulin on DNA synthesis in cultured mouse mammary gland. *Biochim. Biophys. Acta.*, **610**, 235.

JACOBS, M.M., FORST, C.F. & BEAMS, F.A. (1981). Biochemical and clinical effects of selenium on DMH-induced colon cancer in rats. *Cancer Res.*, **41**, 4458.

KINGSNORTH, A.N. (1984). Trace elements in adult total-parenteral nutrition. *Brit. J. Parent. Ther.*, **5**, 8.

PARKER, R.D.R. & SHARMA, R.P. (1978). Accumulation and depletion of vanadium in selected tissues of rats treated with vanadyl sulphate and sodium orthovandate. *J. Environ. Pathol. Toxicol.*, **2**, 235.

RAMASARMA, T. & CRANE, F.L. (1981). Does vanadium play a role in cellular regulation? *Curr. Topics Cell Reg.*, **20**, 247.

SABBIONI, E. & MARRAFANTE, E. (1981). Relations between iron and vanadium metabolism: in vivo incorporation of vanadium into iron proteins of the rat. *J. Toxicol. Environ. Health*, **8**, 419.

SCHWARZ, K. & MILNE, D.B. (1971). Growth effects of vanadium in the rat. *Science*, **174**, 426.

SCOTT, J.F., FRACCASTORO, A.P. & TAFT, E.B. (1956). Studies in histochemistry: I. Determination of nucleic acids in microgram amounts of tissue.. *J. Histochem. Cytochem.*, **4**, 1.

SHARMA, R.P., OBERG, S.G. & PARKER, R.D.R. (1980). Vanadium retention in rat tissues following acute exposures to different dose levels. *J. Toxicol. Environ. Health*, **6**, 45.

SIMONS, T.J.B. (1979). Vanadate – a new tool for biologists. *Nature*, **281**, 337.

SMITH, J.B. (1983). Vanadium ions stimulate DNA synthesis in Swiss mouse 3T3 and 3T6 cells. *Proc. Natl Acad. Sci. (USA)*, **80**, 6162.

WORLD HEALTH ORGANISATION, (1973). Trace elements in human nutrition. *World Health Organisation Technical Report Series No. 532*. Report of a WHO Expert Committee. WHO: Geneva.